# Evaluation kinaesthetic proprioceptive deficit after knee anterior cruciate ligament (ACL) reconstruction in athletes

**DOI:** 10.1186/s40634-019-0174-8

**Published:** 2019-02-07

**Authors:** E. Laboute, E. Verhaeghe, O. Ucay, A. Minden

**Affiliations:** 1 C.E.R.S, Ramsay Générale de Santé, 83 av Maréchal de Lattre de Tassigny, 40130 Capbreton, Capbreton, France; 2 Université Catholique de Louvain, Place P. de Coubertin, 1348 Louvain-la-Neuve, Belgium

**Keywords:** Anterior cruciate ligament, Reconstruction, Proprioception, Kinaesthesia, Reproducibility, TDPM, Sport

## Abstract

**Purpose:**

The objective of this study was to evaluate kinaesthetic proprioceptive deficit after knee anterior cruciate ligament (ACL) reconstruction in two populations of athletes, those in the post-surgery period and those in re-training during the intensive program-training phase.

**Methods:**

We performed a prospective study in ACL-operated athletes without previous knee injuries, with 32 athletes in each group. Time since surgery in the operated athletes in the post-surgery group was 21 to 35 days, and between three and 9 months in the re-training group. We also analysed a control group of 32 uninjured non-operated subjects with a similar sporting level. Proprioception was evaluated using the threshold to detection of passive motion (TDPM) test with Biodex-type isokinetic equipment comparing operated knees, non-operated knees and control uninjured non-operated group. The control group was tested twice, 1 day apart to control reproducibility, using the intraclass correlation coefficient (ICC). The *p*-value threshold for statistical significance between different groups in hypothesis testing was <.05.

**Results:**

TDPM reproducibility was excellent (right knee: ICC = 0.80, left knee: ICC =0.72). We found a bilateral kinaesthetic deficit in post-surgery patients compared to the control group (*p* < 0.001 and *p* = 0.011), which was significantly higher on the operated side (*p* = 0.001). Re-training patients had no significant difference between operated and uninjured knees, but had a kinaesthetic deficit on operated limbs (*p* = 0.036) compared to the control group.

**Conclusion:**

There was a bilateral deficit in post-surgery athletes with a significant difference between injured and healthy knees, which could be explained by a change in the central nervous system. Compared to the control group, a proprioceptive deficit was only seen for re-training patients on the operated side and not in the healthy limb. Kinaesthetic recovery may be faster for the uninjured side as initial deficit is lower.

Level of evidence II.

## Introduction

The anterior cruciate ligament (ACL) is essential for the structural and functional stability of the knee. ACL tears are frequent in sport traumatology, within an incidence in the US of 100,000 cases annually in 2010 (Micheo et al., 2010). ACL reconstruction leads to histological and physiological modifications which have consequences on proprioceptive, muscular and functional sport performances (Ben Moussa Zouita et al., 2008; Zouita Ben Moussa et al., 2009).

Afferent proprioception information comes from articular, muscular and cutaneous receptors. The ACL may have up to 2.5% of neural elements consisting of ruffini nerve endings, Golgi tendon organs (both slow-adapting receptors which are sensitive to joint positioning) and Pacinian corpuscles (fast-adapting receptors, which are sensitive to changes in speed or direction during a joint movement) (Jennings, 1994; Ozenci et al., 2007; Reider et al., 2003; Riemann et al., 2002; Schultz et al., 1984). Using its various receptors and acting as a static stabiliser for the knee, the ACL also plays a dynamic stabiliser role, which allows joint muscular tension adaptation (Boerboom et al., 2008). Studies have suggested that proprioceptive deficiency may be responsible for instability after an ACL tear occurs, even in the absence of any significant motor deficit (Ben Moussa Zouita et al., 2008).

Several studies have reported proprioceptive modifications after an ACL tear both following knee reconstruction (Anders et al., 2008; Angoules et al., 2011; Ben Moussa Zouita et al., 2008; Bonfim et al., 2003) and without surgery (Bonfim et al., 2008; Borsa et al., 1997; Friden et al., 1997; Friden et al., 1999), however others have reported an absence of such modifications (Harrison et al., 1994; Henriksson et al., 2001; Mattacola et al., 2002; Pap et al., 1999), and a consensus is yet to be reached. It is important to keep in mind that these studies include patients with very different post-surgery follow-up durations, which is likely to have a major influence on the level of proprioceptive deficit and rehabilitation. Angoules (Angoules et al., 2011) showed decreased joint position sense (JPS) and threshold to detection of passive motion (TDPM) performances for injured knees both before surgery and 3 months later, but not at six or 12 months post-surgery. Moreover, in most of the studies the time between surgery and enrolment was very long sometimes 2 years (Ben Moussa Zouita et al., 2008; Henriksson et al., 2001; Mattacola et al., 2002), with only a few studies performing proprioceptive evaluation immediately after surgery (Angoules et al., 2011; Reider et al., 2003).

It is also important to highlight protocol differences between these studies, notably with respect to the reference used for evaluating proprioceptive deficit in the injured knee. Some studies use the contralateral limb for comparison (Angoules et al., 2011; Bonfim et al., 2003; Dauty et al., 2010), whereas others use healthy populations (Anders et al., 2008; Denti et al., 2000; Fremerey et al., 2000), which makes comparisons of the outcomes challenging.

In the current study, we thus implemented a double comparison using both the contralateral knee as well as a healthy population. The aim of the study was to analyse kinaesthetic modifications in a population of athletes after ACL reconstruction, and compared them with a population uninjured athletes having a similar sporting level. After verifying the reliability of the TDPM test in this setting, comparisons were made at two time-points, post-surgery (at the end of the first month) and during re-training (when running is possible and before the end of the period of rehabilitation). TDPM was selected as the measure of proprioception deficit, as it is considered the most sensitive and validated test for detecting the start of a movement (Reider et al., 2003).

## Materials and methods

We carried out a prospective study of proprioceptive evaluation using kinaesthetic motion detection after ACL reconstruction in athletes. Reproducibility of the kinaesthetic test was evaluated. And we have measured these kinaesthetic modifications in two operated populations in terms of timing, one during the post-surgery period and the other during the re-training phase. We also compared the results of these injured athletes to a healthy non-operated population. Thus three cohorts of athletes were included, a post-surgical group, a re-training period operated group and a control non-operated group. To be eligible, patients had to have undergone prior ACL reconstruction, be aged between 18 and 40 years old, with a post-surgery delay between 21 and 35 days for the post-surgery operated population or between three and 9 months for the operated population in the re-training phase. The athletes had to have participated in at least regional competitions during the previous 12 months. Patients with previous orthopaedic or traumatic injuries to both knee, simultaneous joint injuries, knee flexion less than 90°, pain, or who were taking any associated medication were excluded.

Proprioception was evaluated using the kinaesthetic test TDPM (Reider et al., 2003) on a Biodex-type isokinetic equipment (Biodex Medical Systems, Shirley, NY). Both knees were evaluated one after another, starting with the uninjured knee to reassure athletes. Athletes were seated with 5 cm between the popliteal fossa and the seat to prevent contact with the underlying surface. Athletes wore shorts to minimize material rubbing against their skin. The thigh was strapped and the leg was connected to the mobile arm of the Biodex using a pneumatic cuff at the level of the ankle. The centre of rotation was placed at the level of the lateral condyle of the knee. For each participant, the anatomic zero point was considered to be the extension position. Athletes were blind-folded and listened to music loud enough to cover any sounds from equipment sound, using headphones, because white noise was impossible with the other patients. The same music was used for all persons. Athletes used a press-button to stop the equipment with a single press of the thumb. Before initiating the TDPM test, a full explanation was given to each patient. Each athlete performed two practice tests (listening to music and blind-folded) to become familiar with the procedure. The starting position of each measurement was a 15° knee flexion (Pitman et al., 1992). After an unspecified period of time, the limb was moved into flexion with a 4°/sec angular velocity. Athletes were asked to use the thumb-button as soon as they detected a knee movement. Pressure on the button instantaneously stopped the movement. The range of motion between the initiation of the movement and the stopping of the machine was recorded in degrees. The examiner then returned the device to the initial position, replacing the leg at 15° flexion, by an extension movement to 0° then a flexion of the knee to 15° which was calculated by a program in the Biodex. At the starting position, the athlete was informed that the next test would begin. The entire TDPM procedure included 10 tests for both limbs. The range of motion between the start position and the perception of movement were averaged for each knee. In first, we checked the reproducibility of this TDPM procedure on an isokinetic Biodex-style machine. In this context, we performed two measures on a healthy control population at a 1 day interval. The study was approved by the Ethics Biomedical Commission of the Academic Hospital of the UCL of Louvain-la-Neuve (Number: B403 2012 14,124). All participants provided written informed consent.

## Statistical analysis

Statistical analyses were performed using the Statistical Package for Social Sciences (IBM SPSS Statistics for Windows, Version 19, Chicago, Illinois) and verified by an expert statistician. Reproducibility was determined using the intraclass correlation coefficient (ICC) for both knees in the healthy non-operated population. The ICC scale was defined as insufficient for < 0.4, medium to good for 0.4–0.7, or excellent for > 0.7. Groups were compared using univariate variance analysis (ANOVA, Tukey post-hoc tests) for mean values after we have verified normality and homogeneity. The *p*-value threshold for statistical significance in hypothesis testing was <.05. For the healthy non-operated population, statistical analyses of knee performances were combined to avoid influencing results by the dominant side (i.e., the mean of the right and left knees). And we have used chi2 and T tests to verify if populations have statistical differences in terms of age, sex, laterality, type of surgery, sporting level, and type of sport.

## Results

### Population

Thirty-two athletes were included in each of the three cohorts (Table 1). The mean time since surgery was 29.4 days for the post-surgery group, and 164.7 days for the re-training group. Mean age was 24.9 years for both operated groups of athletes, and 26.2 years for the control group. All three groups included more male athletes (post-surgery 78.1%; re-training 68.8% and control 68.8%). There were no statistical differences in terms of age, sex and sporting level between the three groups, with similar proportions of patients participating at the regional and national levels, slightly favouring regional sport. Four surgical techniques were used in each of the two operated groups of patients (Table 2). Hamstring tendon autografts were the most common technique in both groups, used for 78.1% of post-surgery athletes and 75.0% of re-training athletes. The second most common technique in both groups was patellar tendon autografts, reported for 12.5% of post-surgery athletes and 15.6% of re-training athletes. Four-strand semitendinosus graft and fascia lata graft (intra and extra articular) were used with the same proportion in both groups. There were no significant differences between groups in terms of the surgical technique used. In the post-surgery group, 17 left knees and 15 right knees were reconstructed. In the re-training group, 16 left knees and 16 right knees were reconstructed. There were no significant differences between groups for injury laterality.

**Table 1:** Socio-demographic descriptive characteristics of athletes

Group	Number of subjects	Mean age in years (±SD)	Sex		Sporting level	
			Men (%)	Women (%)	Regional (%)	National (%)
Post-surgery (operated athlete)	32	24.9 (±5.9)	78.1	21.9	56.3	43.8
Retraining (operated athlete)	32	24.9 (±5.3)	68.8	31.3	62.5	31.3
Control (uninjured athletes)	32	26.2 (±6.2)	68.8	31.3	65.6	31.3
P-value	n.s	n.s	n.s		n.s	

**n.s.:** not significant

**Table 2:** Additional descriptive characteristics of athletes

Group	Surgical technique				Laterality	
	HTA (%)	DT4 (%)	PTA (%)	MIFL (%)	Right (%)	Left (%)
Post-surgery (operated)	78.1	6.3	12.5	3.1	46.9	53.1
RA (operated)	75.0	3.1	15.6	6.3	50.0	50.0

*HTA* hamstring tendon autograft technique, *PTA* patellar tendon autograft technique, *DT4*: 4-strand graft technique, *MIFL*: Fasia Lata transplant

### Reproducibility of the TDPM test

The ICC between the two TDPM tests on two consecutive days in the control non-operated group was 0.80 for the right knee and 0.72 for the left knee, reflecting very high reproducibility for both knees.

### Kinaesthetic evaluation in terms of timing

Kinaesthetic evaluation was performed by comparing the following five sub-groups: operated knees of post-surgery athletes, healthy knees of post-surgery athletes, operated knees of re-training athletes, healthy knees of re-training athletes, and left and right healthy knees of non-operated athletes (Table 3).

**Table 3:** Kinaesthetic sense and statistical analysis of both knees in operated and non-operated athletes

Groups	Mean degrees ± SD	Post-surgery operated knees	Post-surgery healthy knees	RA operated knees	RA healthy knees	Control
Post-surgery operated knees (operated athlete) (*N* = 32)	1.22° ± .53		.001 *	<.001 *	<.001 *	<.001 *
Post-surgery healthy knees (operated athlete) (*N* = 32)	.95° ± .29	.001 *		.995	.855	.011 *
RA operated knees (operated athlete) (N = 32)	.91° ± .35	< .001 *	.995		.974	.036*
RA healthy knees (operated athlete) (N = 32)	.86° ± .34	< .001 *	.855	.974		.160
Control healthy knees (non-operated athletes) (N = 32)	.66° ± .19	< .001 *	.011 *	.036 *	.160	

*RA* the retraining group, *HTA* hamstring tendon autograft technique, *PTA* patellar tendon autograft technique, *DT4* 4-strand graft technique, *MIFL*: fascia lata transplant technique, * Significant difference between the 2 groups (*p* < 0.05)

### Post-surgery patients

A significant difference was seen between knees of the control non-operated group versus the operated knees in the post-surgery group (mean .66° vs 1.22°; *p* < .001; mean difference .56°) as well as versus healthy knees in the post-surgery group (.95°; *p* = .011; mean difference .29°) which might reflect lower bilateral kinaesthetics immediately after surgery. We also identified a significant difference between operated knees (1.22°) and healthy knees (0.95°) in the post-surgery group (*p* = .001; mean difference .27°) which suggests lower kinaesthetic perception for operated knees compared to healthy knees.

### Re-training period patients

In the re-training group, a significant difference (*p* = .036; mean difference .25°) was seen for athletes between operated knees (.91°) and knees in the control non-operated population (.66°). However there was no difference between healthy knees (.86°) and either the operated knees of the retraining group or the healthy knees of the control non-operated group (*p* = .16), reflecting that a kinaesthetic deficit is only present for re-training operated knees.

### Evolution over time in operated versus healthy knees

Interestingly, a significant difference was found between operated knees in the post-surgery (1.22°) and re-training (.91°) groups (*p* < .001; mean difference .31°), which may reflect that the improvement of the kinaesthetic sense of the operated knee depends on the post-surgical delay. For operated knees in the re-training group, there was a significant difference (*p* = .036; mean difference .25°) with the control non-operated group (.66°), supporting incomplete recovery of the kinaesthetic perception in healthy knees even during re-training phase. The healthy knee of the post-surgery group (.95°) was significantly different (*p* = .011; mean difference .29°) from the control group (.66°), but not the healthy knee of the re-training subgroup (.86°). A lower initial deficit after surgery for the healthy knee compared to the operated knee could lead to a slightly faster recovery of the kinaesthetic perception of the healthy knee compared to the injured knee.

## Discussion

We have demonstrated a bilateral kinaesthetic deficit during the post-surgical phase of ACL reconstruction surgery. This bilateralism confirms previous reports in multiple studies, including in patients with a deficient ACL (Bonfim et al., 2008; Gauffin et al., 1990; Lysholm et al., 1998; Pap et al., 1999) and in some cases after ACL reconstruction (Denti et al., 2000; Fremerey et al., 2000). The hypothesis proposed in the literature to explain this bilateralism is a change in the central nervous system (CNS), with the loss of ACL mechano-receptors that occurs during the tear altering the proprioceptive information in the CNS. Supporting this theory are reports of central reorganization after sensory deprivation or a peripheral lesion (Kapreli & Athanasopoulos, 2006). Several studies have reported central somatosensory changes after ACL tears (Courtney & Rine, 2006; Valeriani et al., 1996; Valeriani et al., 1999), and the cerebral cortex is known to participate in voluntary motor control. Pitmann et al. (Pitman et al., 1992) have reported somatosensory evoked potentials after direct stimulation of the ACL during surgery. Valériani et al. (Valeriani et al., 1996; Valeriani et al., 1999) have measured somatosensory evoked potentials before and after reconstruction in seven patients and found decreased knee kinaesthetic position perception and a lack of the cortical P27 potential on the side of the ACL lesion. Baumeister et al. (Baumeister et al., 2008) have also shown that electroencephalography changed cortical activity after ACL reconstruction. Modifications mostly concern the anterior frontal cortex (theta) and parietal cortical (alpha-2) areas. These modifications are also found during stimulation of healthy limbs in operated patients, explaining the change of proprioceptive capacities. This central reorganization could explain bilateral proprioceptive modifications.

Nevertheless, despite a bilateral deficit after surgery, no significant difference between operated and healthy knees was present during the re-training period. Various studies have found no differences between operated and healthy knees 3, 6 and 12 months after surgery (Angoules et al., 2011; Muaidi et al., 2009a; Muaidi et al., 2009b), but have not compared athletes’ performance to a control group. The current study highlighted a kinaesthetic deficit for operated knees during re-training (*p* = .036) compared to non-operated controls. Healthy knees in re-training and non-operated patients gave similar performances (*p* = .160). These results show a likely partial recovery of proprioceptive capacities in injured limbs and almost complete recovery in healthy limbs during re-training. Recovery in injured limbs can be expected to be slower than in healthy (non-injured) limbs. The same principal may apply with a greater kinaesthetic deficit in operated knees than in healthy ones immediately after surgery (*p* = .001). There may be an evolution of proprioceptive capacities during recovery, as both operated and healthy knees in re-training athletes were significantly more efficient in proprioception than operated knees immediately after surgery. It would have been interesting to follow the same population after surgery and re-training to confirm this faster recovery for the healthy knee. Based on these results, it is difficult to compare healthy limbs to injured ones for evaluation during re-training, and it is important to perform proprioceptive rehabilitation for both injured and healthy knees after surgery, at least until the re-training phase.

The TDPM test has often been used with an electrogoniometer (Reider et al., 2003) in the literature. We have confirmed its reliability with the isokinetic Biodex® machine which measures the same movement, with the control group performing two evaluations with a one-day interval. This short interval took into account that athletes would not improve their proprioceptive capacities between the tests. High reliability was seen with an ICC between the two tests of .80 for the right knee and .72 for the left knee. In our study, kinaesthetic capacities were assessed by TDPM, which was selected from the proprioceptive reference tests available in the literature in light of its superior sensitivity and reliability (Ageberg, 2002; Ageberg et al., 2007; Reider et al., 2003). As joint receptors are mostly stimulated at the end of the range of motion, the knee is more sensitive to movement close to complete extension, and proprioceptive deficit after ACL tears are better detected. To account for this, the test began with a 15° flexion of the knee (Ageberg et al., 2007; Angoules et al., 2011; Friden et al., 2001; Friden et al., 1996; Friden et al., 1997; Ozenci et al., 2007; Riemann & Lephart, 2002; Riemann et al., 2002). A slow angular speed was chosen to increase stimulation of joint receptors and decrease the neuromuscular spindle as suggested by some studies (Ageberg et al., 2007; Friden et al., 1997; Ozenci et al., 2007; Riemann & Lephart, 2002), even when using slower angular speeds of between .5 and 2.5°/sec. We decided to slightly increase angular speed (to 4°/sec), to increase the extent of the arc in degrees and emphasize the differences between groups. Concerning detection of a passive motion, we limited input from other information sources with patients being blindfolded, listening to music to ensure any equipment sound was covered, with shorts to minimize skin rubbing sensations with the ankle strapped to the arm of the Biodex.

The use of this test in a research protocol is relevant, but not in clinical valuation because of the small clinical difference (for example .56° between knees of the control non-operated group versus the operated knees in the post-surgery group) despite significant difference (*p* < .001). As a result, this evaluation is not commonly used in rehabilitation protocols. For the future, it could be more important to have a global approach with functional valuation (analyse of running or jumping for example) which is closer to the real-life setting, especially for athletes, despite this approach don’t make difference between proprioceptive and muscular parameters.

## Conclusion

We identified a bilateral proprioceptive deficit after ACL surgery in injured athletes, with a significant difference between operated and healthy knees. A deficit on injured limbs during re-training was only seen compared to the control group but not to healthy limbs. We presume that kinaesthetic recovery is faster for healthy knees than for injured ones. These results also show the difficulty of comparing healthy and injured knees as no differences were identified during re-training. The results must be compared to a control population to be interpretable during rehabilitation. In the future, more ecological procedures will probably be used to assess deficit during re-training such as functional evaluation which is closer to the real world, especially for athletes.

## Abbreviations

ACL: anterior cruciate ligament
ICC: intraclass correlation coefficient
TDPM: threshold to detection of passive motion
